# The Virtual Sleep Lab—A Novel Method for Accurate Four-Class Sleep Staging Using Heart-Rate Variability from Low-Cost Wearables

**DOI:** 10.3390/s23052390

**Published:** 2023-02-21

**Authors:** Pavlos Topalidis, Dominik P. J. Heib, Sebastian Baron, Esther-Sevil Eigl, Alexandra Hinterberger, Manuel Schabus

**Affiliations:** 1 Laboratory for Sleep, Cognition and Consciousness Research, Department of Psychology and Centre for Cognitive Neuroscience Salzburg (CCNS), Paris-Lodron University of Salzburg, 5020 Salzburg, Austria; 2 Institut Proschlaf, 5020 Salzburg, Austria; 3 Department of Mathematics, Paris-Lodron University of Salzburg, 5020 Salzburg, Austria; 4 Department of Artificial Intelligence and Human Interfaces (AIHI), Paris-Lodron University of Salzburg, 5020 Salzburg, Austria

**Keywords:** automatic sleep staging, heart-rate variability, wearables, machine learning, digital CBT-I

## Abstract

Sleep staging based on polysomnography (PSG) performed by human experts is the de facto “gold standard” for the objective measurement of sleep. PSG and manual sleep staging is, however, personnel-intensive and time-consuming and it is thus impractical to monitor a person’s sleep architecture over extended periods. Here, we present a novel, low-cost, automatized, deep learning alternative to PSG sleep staging that provides a reliable epoch-by-epoch four-class sleep staging approach (Wake, Light [N1 + N2], Deep, REM) based solely on inter-beat-interval (IBI) data. Having trained a multi-resolution convolutional neural network (MCNN) on the IBIs of 8898 full-night manually sleep-staged recordings, we tested the MCNN on sleep classification using the IBIs of two low-cost (<EUR 100) consumer wearables: an optical heart rate sensor (VS) and a breast belt (H10), both produced by POLAR®. The overall classification accuracy reached levels comparable to expert inter-rater reliability for both devices (VS: 81%, κ = 0.69; H10: 80.3%, κ = 0.69). In addition, we used the H10 and recorded daily ECG data from 49 participants with sleep complaints over the course of a digital CBT-I-based sleep training program implemented in the App NUKKUAA™. As proof of principle, we classified the IBIs extracted from H10 using the MCNN over the course of the training program and captured sleep-related changes. At the end of the program, participants reported significant improvements in subjective sleep quality and sleep onset latency. Similarly, objective sleep onset latency showed a trend toward improvement. Weekly sleep onset latency, wake time during sleep, and total sleep time also correlated significantly with the subjective reports. The combination of state-of-the-art machine learning with suitable wearables allows continuous and accurate monitoring of sleep in naturalistic settings with profound implications for answering basic and clinical research questions.

## 1. Introduction

Increasingly poor and insufficient sleep is considered a global concern [1] with detrimental public health implications [2]. Persistent sleep disturbances have been related to chronic health diseases, such as cardiovascular and cognitive disease, accidents at the workplace with profound financial consequences, and diminished quality of life [3,4]. As a result, there is rapid growth in the sleep aids market—from USD 74.3 billion in 2021 to around USD 124.97 billion by 2030 [5] with the hope of improving sleep and sleep quality in the general public. Due to methodological constraints, however, the objective assessment of sleep interventions aiming at improving sleep is rarely conducted.

Polysomnography (PSG), a combination of electroencephalography (EEG), electromyography (EMG), and electrooculography (EOG), is scientifically established as the gold standard for the objective measurement of sleep. Based on specific EEG/EOG/ECG criteria human experts characterize every 30-s sleep epoch according to AASM criteria [6] in five classes: wake, rapid eye movement sleep (REM), and increasingly deep non-REM sleep stages (N1, N2, N3). These different sleep stages are also considered to carry different biological functions (e.g., memory consolidation [7], brain waste removal [8], or even emotional information processing [9]) and have been related to the restorative effect of sleep as well as well-being [10]. The manual 30-epoch sleep stage classification provides a useful, fine-grained insight into sleep, which is necessary for the monitoring of sleep interventions, but it comes with several drawbacks: it is rather cost- and time-consuming, the set-up is difficult, and PSG is thus not suitable for daily recordings and/or naturalistic settings (e.g., people’s homes). For this reason, there is the need for a sleep staging procedure that addresses these constraints without sacrificing the fine-grained, epoch-by-epoch, and multi-class nature of traditional PSG.

A solution to this problem involves the combination of alternative physiological signals, such as the cardiac signal, and machine learning approaches for automatic sleep staging [11]. The cardiac signal is an easy-to-obtain signal that comes with a high signal-to-noise ratio, and follows distinct patterns depending on the sleep stage [12,13]. For example, it is well documented that the heart-rate slows down and becomes more regular in NREM sleep [14,15], while in REM it is elevated and more varying [13,16]. Several studies have successfully applied machine learning to cardiac signals and can classify sleep into several stages [17,18]. Li et al. [19] for example used a large dataset with cardiorespiratory coupling spectrograms to train a three-layer convolutional neural network and reported an overall epoch-by-epoch four-class (Wake, REM, NREM light, and deep sleep) accuracy of 75.4% (κ = 0.54). One of the highest classification accuracies was reported by Sridhar et al. [11] who extracted heart-rate time series from ECG and trained a convolutional neural network, with additional layers that modified the network’s field of view, resulting in the overall Cohen’s κ of 0.66 and an accuracy of 77%. It is evident that cardiac signals in combination with machine learning promise to approximate epoch-by-epoch sleep staging accuracies of human experts relying on PSG data.

Another step toward less costly and convenient sleep monitoring would involve the simple use of consumer devices readily available on the market. Many of such wearables claim to acquire beat-to-beat intervals reliably using photoplethysmography (PPG) and relying on the pulse wave [20]. In a systematic review on wearables, Imtiaz [20] identified 35 studies that used the cardiac signal (partly in combination with accelerometers and/or respiration) and validated their sleep stage classification algorithms against PSG. However, only a few studies [21,22,23,24,25,26] recorded the cardiac signal using simple commercial devices and provided accuracy levels for at least four-stage classification as well as a κ metric [27]. Note that a κ metric is necessary as per-chance results can already be in the order of 50% accuracy if a classifier would simply stage “light sleep” throughout the night. The six studies mentioned above report classification accuracies ranging between 64% and 77% with corresponding κs ranging from moderate to substantial agreement (0.48–0.67) [28].

With the use of consumer devices, sleep staging is also made possible on a daily basis and in naturalistic settings. Such devices, if thoroughly tested and validated, could consequently also be used to continuously evaluate the success of sleep intervention programs. With 25% of the western world suffering from bad sleep quality and non-restorative sleep, insomnia is the logical candidate for such sleep training programs. Cognitive Behavioral Therapy for Insomnia (CBT-I) is the first-line treatment for tackling such issues [29] and is meanwhile also 24/7 available using digital alternatives (e.g., digital CBT-I). Digital CBT-I has been shown to have positive and lasting effects [30], even comparable to face-to-face therapy [31]. As a downside, such success usually relies on subjective reports only, with less conclusive results when sleep is measured objectively [32]. However, this effect could also relate to the fact that most studies rely only on single sleep laboratory nights and full PSG montages [32], which might not reveal ecologically valid sleep data, especially in people suffering from bad sleep quality already in natural settings.

The purpose of the current study is thus the evaluation of an automatic sleep staging approach that can be used—with accurate and affordable wearables—for the monitoring of daily changes in naturalistic settings. Importantly, we here rely on only heart-rate variability data and verify the algorithm’s classification performance against manually staged PSG-based 30-second epochs. As proof of principle, we then use that wearable-based sleep classification in a sample of participants with self-reported sleep complaints and monitor their sleep over the course of a 6-week period with the aim to ease insomnia complaints.

More specifically, in the current study, we first trained a multi-resolution convolutional neural network (MCNN) on automatically extracted IBIs from the ECG channels of a large collection of sleep data (*N* = 8898) with various PSG recordings that have been sleep-scored by human experts based on standard AASM criteria. We cross-validated the model on four classes (Wake, Light [N1 + N2], Deep, REM), and then adjusted and tested it in a smaller dataset (*N* = 102) with two low-cost (<EUR 100) consumer wearables, namely (i) an ECG breast belt (POLAR® H10) and (ii) an optical PPG heart-rate sensor (POLAR® Verity Sense, VS). The model performance was then expressed using overall accuracy rates, Cohen’s κ, and correlation coefficients, and visualized with Bland–Altman plots. As proof of principle, we then collected continuous sleep data from 49 individuals at their natural environement (i.e., homes) and over the course of a 6-week online CBT-I sleep training program.

## 2. Methods

### 2.1. Participants

A total of 49 (female = 20) participants took part in the sleep training program. We included people older than 18 years old with no self-reported acute mental and neurological diseases, and able to use their smartphones. In order to capture sleep-related changes, we included participants who both completed the sleep training program up to level 5 with at least 5 days in each level. After applying these filters, the data of 34 (female = 11) participants were analyzed. The mean age was 50.06 (*SD* = 12.02, range = 30–73). Although we had no inclusion/exclusion criteria regarding sleep disorders, we found post hoc that over 84.8% of our sample had a Pittsburgh Sleep Quality Index (PSQI) above 5, with a mean of 9.12 (*SD* = 3.23, range = 5–18), suggesting that our sample largely consisted of individuals with bad sleep quality. Across levels, each participant spent on average 7 (*SD* = 3.24, range = 5–26) days in each level. Note that although an upper limit on the number of days that people spent in each level was not applied, the number of days spent in each level was not significantly different between levels (*F*(5, 110) = 1.7, *p* = 0.141, $\eta_{p}^{2}$ = 0.07). The study was conducted according to the ethical guidelines of the Declaration of Helsinki.

### 2.2. Sleep Training

Sleep training was provided with the smartphone application NUKKUAA™ (Version: beta-version). The NUKKUAA™ App offers CBT-I-based sleep training that includes both psychoeducation and cognitive training as well as daily behavioral exercises. More specifically, the sleep training program consists of short daily exercises including educational videos about sleep, relaxation exercises, cognitive training, sleep recommendations, and short blog posts. The program is divided into 6 modules and can be completed in 6 weeks, as each module can be accomplished in 7 days, with daily engagement. The goal of each module is to educate participants about a given sleep-related topic and teach them how to put theory into practice with dedicated behavioral exercises. Besides the sleep training program, NUKKUAA™ allows for daily reliable objective sleep monitoring by tracking the heartbeat. In the current study, only the H10 sensor (see Materials) was used to capture the sleep training effects objectively, and thus, participants who took part in the sleep training program were instructed to daily use this sensor.

### 2.3. Materials

We used two POLAR® sensors (POLAR® Electro Oy, Kempele, Finland): the H10 chest strap (Model: 1W) as well as the Verity Sense (VS, Model: 4J), an optical (photoplethysmography: PPG) heart-rate sensor, to measure the participants’ heart-rate continuously during sleep. Both sensors are affordable (<EUR 100), light-weighted (app. 60 g), and comfortable to sleep with. According to the manufacturer, the battery life of both devices reaches up to 400 h (i.e., 16 days, when used all day long), which makes it ideally suitable for long recordings. The H10 and VS have been recently validated to assess IBIs and heartbeat during rest and physical exercise with almost perfect accuracy [33,34,35].

We used the NUKKUAA™ App for the sleep training program that recently has been developed as a Spin-Off from the University of Salzburg for Android and iOS mobile devices. During the sleep training study, only the H10 was used to capture sleep objectively. The H10 was connected to the user’s own smartphone and the NUKKUAA™ App via Bluetooth. Participants were instructed to start each recording by pressing the “Go to sleep” button on the home screen just before sleep and stop it accordingly upon awakening in the morning using the “Wake up” button.

### 2.4. Data and Statistical Analysis

**Figure 1:**
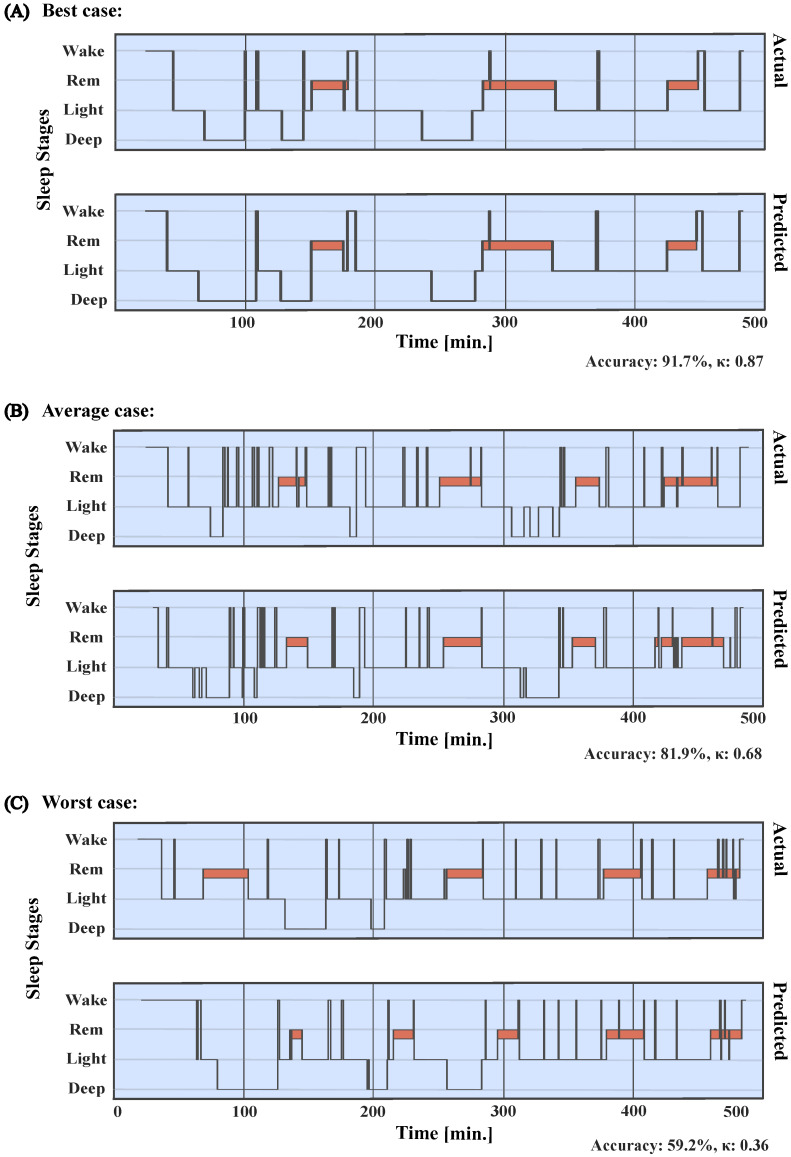
Example of actual and predicted hypnograms for the H10 sensor. The actual hypnograms are sleep-staged based on PSG using the Sleepware G3 software (upper plots), while the predicted hypnograms are based on MCNN sleep stage classification (lower plots) solely trained on the IBIs as extracted from the H10 sensor. We include an example of (**A**) a best classification case (Accuracy: 91.7%, κ: 0.87), as well as (**B**) an average (Accuracy: 81.9%, κ: 0.68) and (**C**) worst case (Accuracy: 59.2%, κ: 0.36) hypnograms.

Participants reported daily when they went to bed and when they woke up, their sleep quality, subjective wake time during the night in minutes, as well as the number of awakenings via the App. For the nights when a sensor was worn, the IBIs were extracted and used for sleep stage classification using the trained model (see the Model Training, Testing, and Performance). Sleep variables were computed for each night based on the hypnograms provided by the MCNN model (see Figure 1 for an example). In order to capture the effects of the sleep training program, we computed the mean of the beginning (weeks 1 and 2) and the end of the program (weeks 5 and 6) per participant. We focused primarily on the following objective sleep variables: sleep onset latency (as defined by the AASM criteria: first wake to first light sleep epoch), sleep efficiency (i.e., percentage of total sleep time/time spent in bed), wake time during sleep (calculated in minutes based on the classified wake epochs during sleep), the number of awakenings (computed by counting how often four consecutive 30-s epochs were classified as wake, i.e., 2 min spent in wake), as well as the minutes spent in each of the 4 sleep stages: Wake, Light [N1 + N2], Deep, REM. In addition, the NUKKUAA™ App provides a sleep score for each night [range 1–10] based on three parameters: subjective sleep quality, lights off regularity, as well as total sleep time. The effect of sleep training on sleep-related variables was statistically examined with a paired-sample *t*-test. Correlation analysis throughout the paper was computed with Spearman’s Rank-Order correlation (denoted as ρ). The agreement was visualized using Bland–Altman plots. Data and statistical analysis were performed in R (R Core Team [36]; version 4).

### 2.5. Model Training, Testing, and Performance Measurement

Based on the work of Sridhar et al. [11], we developed a multi-resolution convolutional neural network (MCNN) that was trained to classify sleep into four stages (Wake, Light [N1 + N2], Deep, and REM) solely using IBIs as input signals. The IBIs were extracted from the ECG channels of a set of 8898 full-night ambulatory and lab PSGs (Large PSG Dataset) from a heterogeneous sample including healthy individuals as well as a subclinical population, which have been manually sleep-staged by human experts based on standard AASM criteria (satisfying the minimum setup according to AASM [6]: bipolar electrooculogram, bipolar electromyogram, as well as electroencephalography from C3 and C4 derivations versus the contralateral mastoid electrode). The mean age in this sample was 65.3 years (*SD* = 10.9, range = 39–94) of which 4779 were female. For the IBI extraction, we first padded each recording to a constant length of 1440 30-s epochs (i.e., 12 h), and applied a 5 Hz FIR (with windowed-sinc) high-pass filter before R-Peak detection. We then automatically detected the IBIs using the PhysioNet Cardiovascular Signal Matlab Toolbox [37]. The IBI signals were pre-processed using the PhysioNet toolbox’s ‘RRIntervalPreprocess’ function with default settings (IBIs smaller than 0.3 and larger than 2 s were linearly interpolated). Finally, the automatically extracted IBIs were upsampled to a constant sampling rate of 2 Hz, and z-transformed. The MCNN did not use any pre-calculated HRV features and it was thus trained on the solely “raw” IBI time series. The MCNN architecture includes an adaptive feature recalibration layer [38], a bidirectional long short-term memory layer as well as temporal context encoders based on multi-head attention modules [39] with relative positional weights [40]. The training of the MCNN was performed in TensorFlow, Python [41]. After training the MCNN on the IBIs of the large PSG dataset, the model was tested using four-fold cross-validation (75% training, 25% testing). For every 30-s epoch, the trained MCNN generated four probability values, one for each class. The class with the highest probability is then used to describe each epoch and produce a hypnogram.

Transfer learning (with no fixed model weights) was then used in order to adjust the model to a smaller dataset (Wearables Dataset), which included PSG and time-synchronized IBIs from the two wearable sensors, H10 and VS, in parallel, in a sample of 102 healthy participants with mean age of 41.2 (*SD* = 12.7, range = 20–74) and average PSQI of 5.6 (*SD* = 3.6, range = 2–18). After transfer learning, the model was tested using the same cross-validation procedure as before for each sensor, respectively. The PSG data in the Wearables Dataset was sleep staged automatically with the Sleepware G3 sleep staging software (Version 4.0.0, Philips; Pennsylvania, PA, USA). Sleepware G3 is an artificial intelligence auto-staging software that is widely used. It has been shown to be non-inferior to manual human staging and can be readily used without the need for manual adjustment [42]. We additionally tested this by comparing the G3 Sleepware to an expert sleep scorer on a sample of 49 in-house recordings and observed indeed a very high κ value of 0.86 between the human scorer and the G3 Sleepware (in four classes). Figure 1 displays examples of the best, average, and worst cases of an actual hypnogram, sleep staging with PSG data and the G3 Sleepware, and predicted sleep staging by the MCNN on the automatically extracted IBIs using the H10 sensor.

We assessed the overall model performance, by computing mean fold classification accuracy, Cohen’s κ [43], and four-class confusion matrices. The relationship between the PSG and MCNN sleep variables was evaluated using Spearman’s rank correlation coefficients (denoted as ρ). Having observed excellent G3 Sleepware sleep staging performance, we visualized the agreement between the G3 sleep-staged PSG and MCNN using Bland–Altman plots [44].

## 3. Results

### 3.1. Model Performance

**Figure 2:**
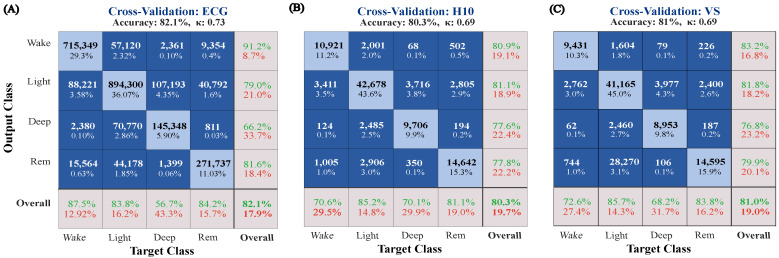
Confusion matrices. (**A**) The MCNN was cross-validated after training on automatically extracted IBIs of the large and manually sleep-staged dataset. The same algorithm was adjusted and cross-validated ON the small dataset with the two wearable sensors (**B**) H10 and (**C**) VS. The rows correspond to the predicted class (output class) and the columns correspond to the true class (target class). The diagonal cells correspond to observations that are correctly classified, whereas the off-diagonal cells correspond to incorrectly classified observations. Both the number of observations and the percentage of the total number of observations are shown in each cell. The gray squares on the right of each matrix include the precision (positive predictive value) and false discovery rates while the ones at the bottom display the recall (or true positive rate) and false negative rate, respectively. The cell in the bottom right corner shows the overall classification accuracy.

**Figure 3:**
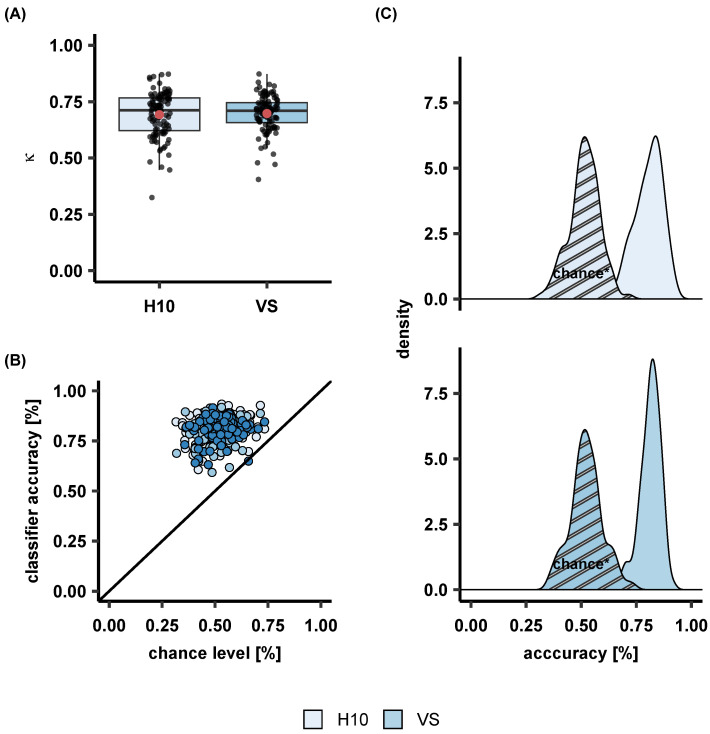
MCNN performance on classifying IBIs against automatic PSG sleep staging as expressed with (**A**) κ values, and (**B**) classification accuracies that are visualized also using (**C**) accuracy density plots with corresponding chance levels* for both low-cost sensors H10, and VS. * The chance level is computed by sleep staging only light sleep which appears to be enough to reach 50% accuracy. Note that sleep staging only with light sleep is sufficient to achieve an accuracy of around 50%.

Overall, cross-validated epoch-by-epoch accuracy on the large PSG dataset was 82.1% (κ = 0.733). Similar performance was observed in the wearables dataset: H10 classification accuracy reached 80.3% and a κ of 0.69 while the VS displayed an accuracy of 81% and a κ of 0.69. Figure 2 illustrates per-class classification true and false positives when the MCNN was trained and tested on the Large Dataset, but also after transfer learning on the Wearables Dataset. Figure 3 displays the performance of the MCNN to classify sleep on IBIs as extracted by the low-cost sensors, reflected in κ values and classification accuracies.

**Figure 4:**
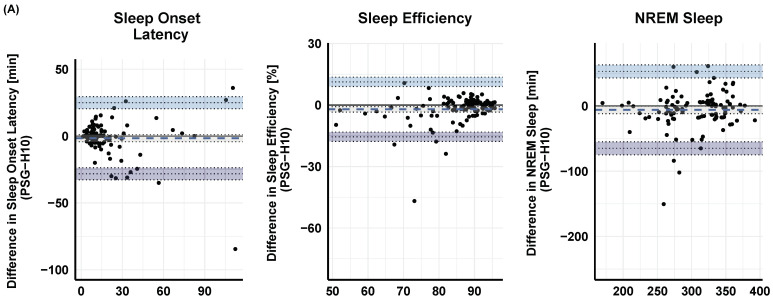

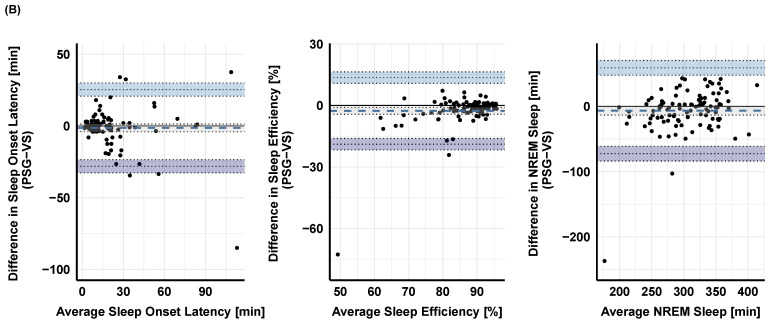
Bland–Altman difference plots between the PSG-based sleep variables for (**A**) H10 (upper row) and (**B**) VS sensor (lower row) on sleep onset latency, sleep efficiency, and NREM (light + deep) sleep. The dashed lines represent the mean difference (i.e., bias) between the two measurements. The dotted line with light and dark blue shading marks the 95 % CI limit of the mean difference. The black solid line represents the point of equality (where the difference between the two devices is equal to 0).

**Figure 5:**
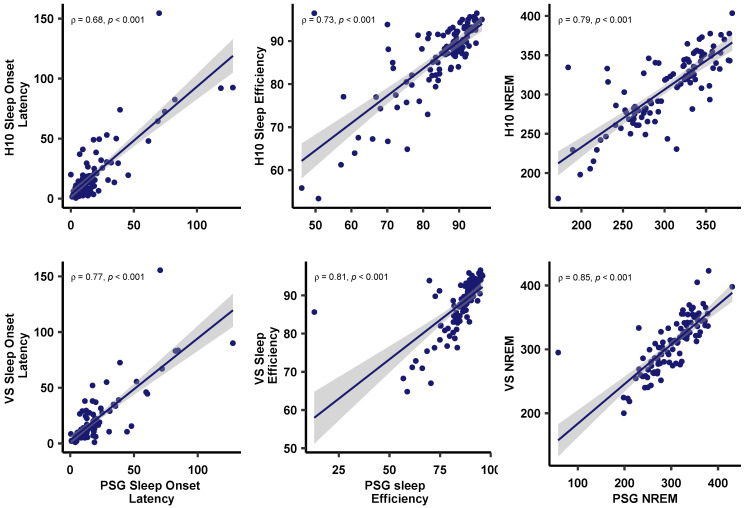
Spearman’s rank correlations ρ between PSG and both (**A**) H10 (upper row), as well as (**B**) VS (lower row) on sleep onset latency, sleep efficiency, and NREM (light + deep) sleep. Individual points reflect sleep parameters as extracted from each device, with the corresponding liner model (line) and 95% confidence interval (shade). Note the slightly higher correlation coefficients for the VS compared to H10.

The agreement between PSG and MCNN-derived sleep onset latency, sleep efficiency, and NREM sleep is visualized in Figure 4 using BA plots. In addition, the PSG was positively and significantly correlated with H10-derived sleep onset latency ρ = 0.68, *p* < 0.001), sleep efficiency ρ = 0.73, *p* < 0.001), and NREM sleep ρ = 0.79, *p* < 0.001). Similar correlation coefficients were observed between PSG and VS: sleep onset latency ρ = 0.77, *p* < 0.001); sleep efficiency ρ = 0.81, *p* < 0.001); NREM sleep ρ = 0.85, *p* < 0.001)—cf. Figure 5.

### 3.2. The Effects of Sleep Training on Subjective and Objective Sleep Variables

**Figure 6:**
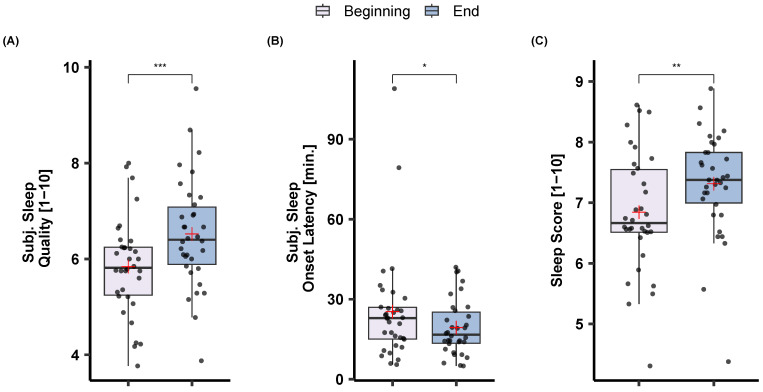
The effects of sleep training on (**A**) sleep quality, (**B**) sleep onset latency, as well as (**C**) sleep score. Sleep score is a mixture of subjective and objective measures (see Methods). Note that participants reported better sleep and shorter sleep onset latency at the end of the sleep training program. *p* < 0.05 *, *p* < 0.01 **, and *p* < 0.001 ***.

**Figure 7:**
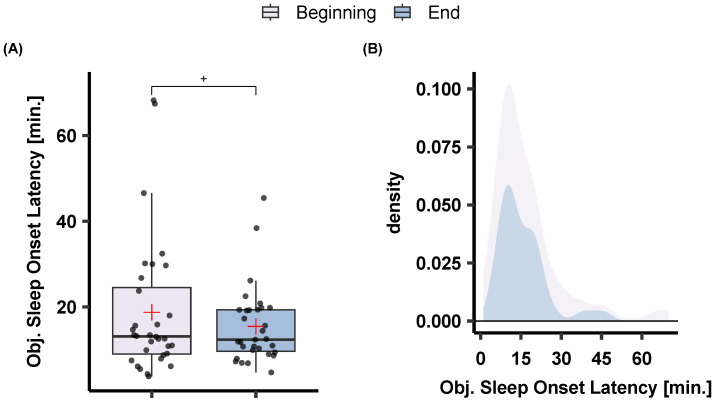
The effects of sleep training on (**A**) objective sleep onset latency as extracted after MCNN sleep classification and (**B**) the respective density plot. Note a trend for a decrease in sleep onset latency at the end of the sleep training program. *p* < 0.1 +.

The effects of sleep training on sleep score and subjective sleep measures are illustrated in Figure 6. An effect of sleep training was observed on sleep score, as it became better with the sleep training time (Beginning: *M* = 6.84, *SD* = 0.98; End: *M* = 7.31, *SD* = 0.86). Sleep training had a significant and positive effect on subjective sleep quality (*t*(33) = −5.39, *p* < 0.001) with participants reporting higher sleep quality at the end of the program (*M* = 6.52, *SD* = 1.13) compared to the beginning (*M* = 5.84, *SD* = 1.01). Similarly, participants reported short sleep onset latency at the end of the sleep training (*M* = 19.38, *SD* = 10.37) compared to the beginning (*M* = 25.35, *SD* = 20.12), *t*(33) = 2.212, *p* = 0.034. The same direction pointed to the objective sleep onset latency as there was a trend for lower objective sleep onset latency at the beginning (*M* = 18.75, *SD* = 16.15) compared to the end (*M* = 15.46, *SD* = 8.93) of the sleep training program, *t*(28) = 1.77, *p* = 0.086 (cf. Figure 7).

**Figure 8:**
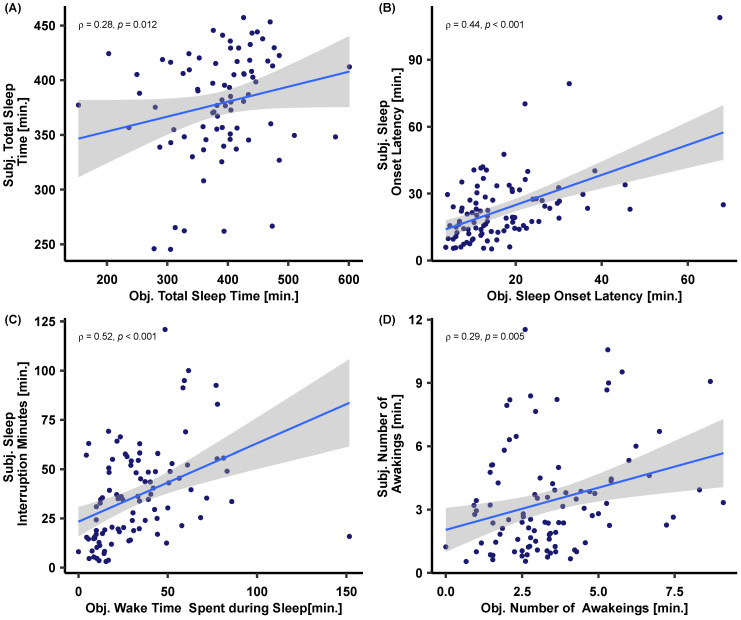
Spearman’s rank correlations (ρ) between subjective and objective (**A**) total sleep time, (**B**) sleep onset latency, (**C**) sleep interruption minutes, as well as (**D**) number of awakenings. Note that individual points correspond to weekly averaged sleep variables per subject.

In addition, as participants estimated their total sleep time, total sleep interruption minutes, and number of awakenings, after every night, we examined the relationship between their subjective reports and these objectively estimated sleep variables. Subjective and objective measures of total sleep time were positively correlated with the objective total sleep time ρ = 0.28, *p* = 0.012) as well as sleep onset latency ρ = 0.44, *p* < 0.001). Similarly, subjective and objective sleep interruption measures were also positively correlated when indexed through both minutes ρ = 0.52, *p* < 0.001) and number of awakenings ρ = 0.29, *p* = 0.004)—cf. Figure 8.

## 4. Discussion

Overall, the results show that our deep learning algorithm—which solely relies on the IBI time series—achieves an accuracy comparable to human expert sleep staging based on PSG data. More specifically, it is known that human experts agree in about 82.6% (five classes, [45]) or 88% (four classes, [45,46]) when staging PSG data. With 81% accuracy (κ = 0.69), we here reached comparable accuracy just by using IBIs, that is a relative agreement of 92% with respect to human four-class sleep staging. In addition, our sleep training proof-of-principle study shows that our trained model together with the chosen wearables (H10 or VS) allow for accurate and affordable daily sleep monitoring epoch-by-epoch.

After training an MCNN on automatically extracted IBIs of 8898 manually sleep-staged PSG nights, we observed high classification performance after 4-fold cross-validation at 82.1% (κ = 0.73). Interestingly, the MCNN showed similar classification accuracies when the IBIs were extracted from the low-cost wearables H10 (80.3%, κ = 0.69) and VS (81%, κ = 0.69). The average κ of 0.69 for the H10 and VS in the present study can be interpreted as substantial agreement on an epoch-by-epoch level, which is similar to the human inter-rater agreement. In a recent meta-analysis by Lee et al. [47], the human inter-rater agreement was estimated at a Cohen’s κ of 0.76, corresponding to 0.70, 0.24, 0.57, 0.57, 0.69 for W, N1, N2, N3, and REM stages). The reason we here combined N1 and N2 as “light” sleep is based on exactly this fact, namely the overall poor inter-rater agreement for N1 sleep. In addition, it is interesting to note that human expert scorers demonstrate sleep staging ambiguity in up to 70–80% of epochs if one takes a close look at multiple scorer agreements [42].

Although we did not define limits of agreement a priori [48], we observed biases for our sleep variables of interest close to 0. Bland and Altman [44] suggest that 95% of data points should lie within ±2 standard deviations of the mean difference; this is what we observe and illustrate for sleep onset latency, sleep efficiency, and NREM (light + deep) sleep (cf. Figure 4). It has been previously discussed that the reason behind the low classification accuracies and κ values in studies that classify based on PPG is an overestimation of N3 sleep [20]. In our data (in the VS sensor), we do not find this but rather observed a small and consistent underestimation of overall NREM sleep (min) as visualized in Bland–Altman plots (cf. Figure 4B). These sleep variables also correlate well when extracted with the gold-standard PSG values and both low-cost devices (cf. Figure 5).

In comparison to previous studies using wearables and cardiac signals [20], our study provides notably higher classification accuracies and κs for both wearable devices. Most previously published studies that used consumer devices have included small and rather homogeneous datasets and/or basic machine learning models. For instance, Hedner et al. [21], used an undetermined model (trained on *N* = 30 + 49 [49,50]) and report low accuracy and Cohen’s κ (i.e., 65.3%, 0.48). Beattie et al. [22] compared different classification approaches (linear discriminant classifiers, quadratic discriminant classifiers, random forests, and support vector machine approaches) and reported that the best classification performance was observed with linear discriminant classifiers that show 69% accuracy and a κ of 0.52. A linear discriminant classifier was used also by Fedorin et al. [24] who reports among these studies the highest accuracies and κ coefficient (77%, 0.58) using a consumer device. In general, it appears that many previous studies with presumably no direct access to a sleep laboratory used rather small datasets for training (e.g., *N* = 12 [25]; *N* = 50 [24]; *N* = 20 [26]). Given the distinct heterogeneity of sleep architectures across the adult population, it is no surprise that models trained on too few examples do not generalize well and consequently lack satisfactory accuracy levels. In addition, shallower machine learning models may not be able to capture all relevant features that are informative for the given classification problem and/or might be not adequately capable of learning the macroscopic architecture of human sleep. Hence, we decided to use a large, heterogeneous training dataset (in age as well as sleep quality measures) together with an advanced deep learning architecture that obtains the entire sleep recording as input. Thereby, our architecture has the ability to extract relevant short (several seconds) and long-term (dynamics up to several minutes) features from sleep recordings and thereby mimic the way human experts score PSGs.

Compared to studies that used deep learning approaches and gold standard ECG recordings before, we also observed slightly higher accuracies (for instance compared to Li et al. [19]: 75.4%, κ = 0.66, and Sridhar et al. [11]: 77%, κ = 0.54). Although our MCNN architecture is based on Sridhar et al. [11], we further included temporal context encoders based on multi-head attention modules [39] to even better capture the temporal profile of sleep staging (i.e., in sleep staging the N − 1 epoch carries information about the N and N+1 epochs [51]). Recently, Habib et al. [52] reported a PPG-based four-stage classification of 93.2% but only after a data augmentation procedure (i.e., 65% before data augmentation) without reporting on generalization abilities (e.g., to other types of wearables). As shown, our model performs equally well for an ECG-based wearable (H10) and a PPG-based wearable (VS). Interestingly, and to our best knowledge, the presented MCNN performs better than published approaches that additionally also include respiratory measures or body movements (κs = 0.5–0.6, accuracies = 69–75.9% ) [19,53,54].

For clarification, we opted for the POLAR® H10 and Verity Sense (VS) sensors after extensive in-house testing of multiple sensors that (i) allowed to extract ECG or PPG data, (ii) were able to store data locally, (iii) gave access to raw inter-beat interval data in the millisecond range and (iv) assumed affordable to the average user (<EUR 100). Applying all these criteria and testing for accuracy against the ECG gold standard in our sleep laboratory, we identified the H10 and VS as the most accurate and suitable consumer devices for our purpose. It is not our aim here to perform a validation of these devices against gold standard ECG but refer the interested reader to recently published validations of that kind [33,34,35].

In addition to the development of our MCNN sleep-staging approach and the selection of suitable consumer devices for the easy sensing of cardiac activity, we also performed a proof-of-principle study using the NUKKUAA™ App. More specifically, we included 49 participants of which 34 completed at least the first 5 levels of the 6-week sleep training program. The sleep training in the NUKKUAA™ App includes classical CBT-I content and advances participants to the next “level” once they fulfil certain criteria, such as performing daily sleep diaries and doing daily CBT-I exercises. In the current proof-of-principle study the App was still in development and participants used a late beta version of the App.

In the current sleep training study, we observed a significant effect on participants’ subjective sleep experience as reflected in higher subjective sleep quality, a reduced subjective sleep onset latency, and an overall higher sleep score, which is a measure combining sleep onset regularity, total sleep time, and subjective sleep quality (cf. Figure 6). We further found a trend towards decreased objective sleep onset latency from the beginning to the end of the program (cf. Figure 7), but not for other objective variables. It is important to note, that in the field it is well-documented that capturing sleep-related intervention benefits via objective measures is much more difficult than finding subjective changes. In a meta-analysis, Mitchell et al. [32] reviewed studies that investigated the effects of dCBT-I on PSG-defined sleep variables and concluded that there actually is no evident beneficial effect. However, Mitchell et al. [32] also noted that the number of studies that could be reviewed and that included PSG was rather small (*N* = 5), with a maximum of one or two nights around the interventions per participant. This is related to the fact that deriving sleep variables with PSG is time-consuming and costly and no longer recordings of objective sleep data are available in such intervention studies. In the current study using easy-to-wear consumer devices, we could work with data from multiple nights per participant and over the course of several weeks.

Using such devices, we found that subjective estimations of sleep duration and sleep fragmentation were indeed correlated with the respective objective measures when averaging over the training weeks (cf. Figure 8). More specifically, we found good agreement between subjective and objective sleep onset latency, and between subjective and objective time spent awake during sleep (defined as wake periods longer than 2 min after sleep onset). Moderate (yet still acceptable) agreement is found between the subjective and objective number of awakenings during the night and the total sleep time. It is worth mentioning that some of the variances here come from known sleep misjudgments of people suffering from insomnia, which has been well documented [55]. Still, we believe it is a valid first sanity check on how far subjective and objective sleep parameters match in such continuous and extended data sets.

Although we believe that the current study contributes substantially to the literature in that it takes objective measures for sleep interventions into account, there are several limitations that need to be considered. First, although we observed a strong and positive effect of the sleep training program on subjective sleep quality, we cannot exclude at this point that such effects are rather unspecific. Future study protocols should include a control group in order to ensure that subjective sleep improvements are indeed attributed to the sleep training program. In addition, future studies that use HRV for sleep staging should examine in more detail how factors such as age, gender, smoking, alcohol consumption, physical activity, or medication influence the HRV metrics and, consequently, sleep staging classifiers. The fact that some of these controls were missing from the current study (only age and sex were considered in the current model) somewhat limits the conclusion. Possibly the lack of objective effects on sleep in our sleep training study results from unspecific variance added by such uncontrolled factors.

## 5. Conclusions

We report on the high epoch-by-epoch sleep classification accuracies and κ in four classes (Wake, Light, Deep, and REM sleep), using solely the inter-beat-intervals from two affordable consumer devices (POLAR® VS: 81%, κ = 0.69; POLAR® H10: 80.3%, κ = 0.69). We observe higher classification accuracies and κ than previous studies that used wearable devices and similar advanced machine learning approaches. We attribute this to the adopted state-of-the-art classification approach in combination with using sufficiently large and heterogeneous training dataset as well as validated consumer devices that allowed accurate extraction of IBI time series. As a proof of principle, we used this sleep staging approach to examine the effects of a novel online sleep training program called NUKKUAA™ and observed correlations between daily subjective and objective measures of sleep length and sleep fragmentation. Our study, thus, contributes to the existing literature by combining advanced machine learning techniques together with low-cost consumer devices that promise accurate and practically daily sleep staging in naturalistic settings and for a multitude of purposes in basic and clinical research.

## Data Availability

Some of the datasets presented in this study are not publicly available due to the fact that they were accumulated over many years and informed consent and ethics approval for sharing the data were not obtained at the time of data collection.

## Abbreviations

The following abbreviations are used in this manuscript:

IBI	Inter-beat Intervals
MCNN	multi-resolution neural network
